# Human macrophage foam cells degrade atherosclerotic plaques through cathepsin K mediated processes

**DOI:** 10.1186/1471-2261-10-19

**Published:** 2010-04-21

**Authors:** Natasha Barascuk, Helene Skjøt-Arkil, Thomas C Register, Lise Larsen, Inger Byrjalsen, Claus Christiansen, Morten A Karsdal

**Affiliations:** 1 Nordic Bioscience A/S, Herlev, DK-2730, Denmark; 2 Wake Forest University School of Medicine, Department of Pathology, Section on Comparative Medicine, Medical Center Boulevard, Winston-Salem, NC 27157, USA

## Abstract

**Background:**

Proteolytic degradation of Type I Collagen by proteases may play an important role in remodeling of atherosclerotic plaques, contributing to increased risk of plaque rupture.

The aim of the current study was to investigate whether human macrophage foam cells degrade the extracellular matrix (ECM) of atherosclerotic plaques by cathepsin K mediated processes.

**Methods:**

We 1) cultured human macrophages on ECM and measured cathepsin K generated fragments of type I collagen (C-terminal fragments of Type I collagen (CTX-I) 2) investigated the presence of CTX-I in human coronary arteries and 3) finally investigated the clinical potential by measuring circulating CTX-I in women with and without radiographic evidence of aortic calcified atherosclerosis.

**Results:**

Immune-histochemistry of early and advanced lesions of coronary arteries demonstrated co-localization of Cathepsin-K and CTX-I in areas of intimal hyperplasia and in shoulder regions of advanced plaques. Treatment of human monocytes with M-CSF or M-CSF+LDL generated macrophages and foam cells producing CTX-I when cultured on type I collagen enriched matrix. Circulating levels of CTX-I were not significantly different in women with aortic calcifications compared to those without.

**Conclusions:**

Human macrophage foam cells degrade the atherosclerotic plaques though cathepsin K mediated processes, resulting in increase in levels of CTX-I. Serum CTX-I was not elevated in women with aortic calcification, likely due to the contribution of CTX-I from osteoclastic bone resorption which involves Cathepsin-K. The human macrophage model system may be used to identify important pathway leading to excessive proteolytic plaque remodeling and plaque rupture.

## Background

Worldwide, cardiovascular disease (CVD) is the leading cause of morbidity and mortality[1]. Atherosclerosis, the underlying cause of CVD events, is a complex and lifelong process which leads to the development of intimal fatty streaks and more complex lesions which may go unnoticed for decades prior to clinical events such as a myocardial infarction. The main event leading to clinically significant disease often involves the rupture of unstable atherosclerotic lesions.

Collagen turnover is mediated by both matrix metallo-proteinases (MMPs) and cathepsins[2]. MMPs have hereto been considered to be the proteases of paramount importance in the atherosclerotic plaques[3,4], however recent research has identified other proteases such as cathepsins and aggrecanasess [5-17] as equally important. This emerging line of evidence demonstrating that extensive proteolytic activity of different origins is of paramount important of the pathogenesis of atherosclerosis, further emphasis the need for understanding the role of the proteolytic array of enzymes in the remodeling of the extracellular matrix of the atherosclerotic plaques. Cathepsin K is a lysosomal protease predominantly secreted by activated macrophages[18] and osteoclasts[19,20]. Cathepsin K has been identified in atherosclerotic plaques [21] and in differentiated macrophages[5-7,11], such as epithelioid cells and multinucleated giant cells in soft tissues[22]. Moreover, disruption of the cathepsin K gene reduces atherosclerosis progression suggesting the proteolytic activity of cathepsin K to be important for the pathogenesis of atherosclerosis[10].

Tissue turnover may be assessed by biochemical markers of tissue degradation[23,24]. Biochemical markers of tissue turnover are increasingly used in both basic and clinical research, for diagnostic, prognostic and efficacy purposes[24,25]. In addition, such markers may provide additional information for understanding the pathology of disease. Proteolytic degradation of ECM molecules by proteases such as cathepsins and matrix-metallo proteinases results in the generation of small protein degradation fragments, neo-epitopes. The collagen turnover in the plaques is primary caused by an increased degradation of collagen type I, which accounts for approximately 60-70% of total collagen in artery[26]. Cathepsin K is the major protease of osteoclasts, responsible for bone resorption[27]. The protease activity of cathepsin K on collagen type I results in a specific degradation fragment, CTX-I (C-terminal telopeptide of collagen type I)[23,28]. This fragment has been extensively used as a surrogate measure of bone resorption for in vitro, preclinical and clinical studies[23,29].

The aim of the current studies was to investigate whether macrophages degraded the articular matrix through processes involving cathepsin K and collagen type I. We examined 1) The expression and localisation of cathepsin K in atherosclerotic plaques, 2) the ability of human macrophage foam cells to generate CTX-I fragments in culture and finally 3) to investigate whether these molecular processes were of clinical significance, we assessed the relationship between the circulating levels of CTX-I molecules and aortic calcification in patients with radiological detectable aortic calcifications.

## Methods

### Sample material for immune-histochemistry

Arterial tissues from two subjects with and without a history of CVD were investigated by immune-histochemistry. Samples were collected 0.15-20 cm distal to the origin of the left descending coronary artery (LAD). The first subject (subject A) was a 77 yr female with an advanced atherosclerotic plaque (cross-sectional area of plaque size was 6.2 mm^2^). The second subject (subject B) was a 32 yr female with a minimal atherosclerotic lesion of 0.2 mm^2^. Sample collection and handling was performed post-mortem in accordance with the guidelines of the medical ethical committee of the Wake Forest University Health Sciences as previously described[30].

### Serum samples

For the clinical investigation, subjects were selected from a previously reported epidemiological study of 1356 elderly women aged 60 to 85 years examined at the Center for Clinical and Basic Research, Denmark[31]. Briefly, the participants in the present study were recruited by questionnaire surveys. In 2000 to 2001, these women were invited for a follow-up examination. All women gave informed consent to participation, and the study was carried out in accordance with the Helsinki Declaration II and the European Standards for Good Clinical Practice. The local ethics committee approved the study protocol. Participants in the present investigation were 102 women, including 51 women diagnosed with CVD and 51 age-matched controls. The CVD diagnosis was based on the presence of calcified deposits in the lumbar aorta visualized on lateral radiographs and graded with scores ranging from 0 to 24. Calcified deposits in the lumbar aorta adjacent to each lumbar vertebra (L1-L4) were assessed separately for the anterior and posterior wall of the aorta using the midpoint of the inter-vertebral space as the boundaries. Severity of aortic calcifications was described by 3 scores: the affected segments score (scale 0 to 4), the anterior and posterior affected score (scale 0 to 8), and the anteroposterior severity score (scale 0 to 24)[32]. Women graded with a score of 8 or more were classified as having CVD in the present investigation. Additional CVD risk factors were collected. Body weight and height were measured and BMI calculated. Arterial blood pressure was measured with a digital blood pressure monitor (UA-777, A&D Instruments LTD). Total cholesterol and triglyceride levels were measured by a Vitros-250 automatic blood analyzer (Johnson & Johnson). All serum samples were collected in the morning from fasted individuals. Demographic characteristics in the study population are indicated in Table 1.

**Table 1:** Characteristics of the study population stratified into women with CVD and the control group.

	Control (n = 51)	Disease (n = 51)	p-value
Age (years)	73.6 ± 0.6	73.5 ± 0.6	0.9079

Triglyceride (mmol/L)	1.35 ± 0.07	1.72 ± 0.13	0.0158 *

BMI (kg/m^2^)	26.8 ± 0.5	26.7 ± 0.5	0.8222

Systolic BP (mm Hg)	148.9 ± 3.8	147.3 ± 3.2	0.7547

Diastolic BP (mm Hg)	82.0 ± 1.7	78.2 ± 1.6	0.1087

Total cholesterol (mmol/L)	6.53 ± 0.14	6.13 ± 0.20	0.1052

Aortic calcification	0	12.6 ± 0.51	

Results shown are mean ± SEM unless otherwise indicated.

### Immunohistochemistry

Hearts were perfusion fixed at 100 mm Hg with formalin, coronary arteries were isolated, blocks were paraffin embedded, sectioned into 5 μM sections and mounted on glass. Pretreatment of sections in buffer 1 (10 mM Tris, 0.25 mM EDTA, pH 9.0) was used to enhance epitope presentation. The sections were blocked for nonspecific binding in Tris buffered saline (TBS) containing 5% casein before incubation with a monoclonal antibody to human CTX-I (in-house) or mouse anti-human Cathepsin K (Chemicon, MAB3324 directed against a synthetic peptide corresponding to amino acids 182-195 of human cathepsin K) or rabbit polyclonal antiserum LF-9 against Bovine alpha1(I) amino-propeptide of Collagen I with cross-reactivity to human, monkey pig and sheep (Larry W Fisher, Matrix Biochemistry Section, Cranofacial and skeletal diseases branch, NIDCR/NIH, Bethesda, Maryland). Peroxidase-labeled mouse or rabbit Envision (DAKO cytomation, DK) was used as secondary antibody. Immunoreactivity was visualized by liquid DAB substrate chromogen solution (3,30-diaminobenzidine chromogen). The nuclei were counterstained using hematoxylin (DAKO), and the slides were dehydrated, cleared and cover-slipped. Digital histographs were taken using an Olympus BX60 microscope and an Olympus CP71 camera.

### Cell culture

Isoltion of CD14+ primary monocytes was performed using coated Dynabeads M-450 (111.11 Dynal Biotech). Human monocytes were isolated from peripheral blood from healthy female volunteers 18 to 45 years old. The blood was diluted 1:1 with PBS (BE17-512F Biowhittaker), carefully layered on a Ficoll-Paque gradient (17-1440-03 Amersham Pharmacia) and centrifuged at 2000 rpm for 20 minutes. The lymphocytes were collected from the interface between the plasma and the Ficoll-Paque, washed with ice-cold PBS followed by centrifugation at 2000 g rpm for 12 minutes. The wash was repeated twice, after which the cells were re-suspended in cold PBS containing 2% serum (S0415 Biochrom). The cells were kept on ice while the beads were prepared. 125 μl CD14+ magnetic beads (10 million beads) were washed 3 times in ice cold PBS. The beads were placed in the magnetic device for 2 minutes between each wash.

Three hundred million cells were added to the beads and incubated at 4°C with end-over-end homogenization for 20 minutes. Hereafter, the beads were washed 5 times in 5 ml PBS containing 2% serum with gentle re-suspension between each washing step. Finally, the cells were re-suspended in phenol-red free alpha-MEM containing 10% serum, 100 units/ml penicillin and 100 μg/ml streptomycin, as previously described[20,33].

To generate human monocyte-macrophage cultures, the CD-14 positive cells were cultured in 75 cm^2 ^bottles in phenol-red free alpha-MEM containing 10% serum, 100 units/ml penicillin, 100 μg/ml streptomycin, 25 ng/ml M-CSF for 3 days. Cells were released using trypsin and replated on 48,8% Matrigel^®^Basement Membrane Matrix (cat.no 40234, lot no. 900618) from Engelbreth-Holm-Swarm (EHS) mouse tumor rich in ECM proteins. Major component of the Matrigel is laminin, followed by collagen IV, heparin sulfate proteoglycan, entactin and nidogen. In our experiments, Matrigel was enriched with 41,5% type I collagen (3 mg/mL), 4,9% 10 × alpha-MEM and 4,9% reconstitutions buffer. After one day allowing the cells to incorporated into the matrix the cells were incubated in either absence (macrophages) or presence (foam cells) of 2 mg/ml LDL (L8292 Sigma-Aldrich) and M-CSF, and with or without selective cysteine protease inhibitor E64 (Calbiochem). Following the culture, cells were fixed in 4% formaldehyde, and stained for lipid droplets by oil red staining. Medium was collected for assessment of the presence of CTX-I indicative of cathepsin K mediated type I collagen degradation.

### Biochemical Analysis

Serum samples were analyzed for degradation fragments of the C-telopeptides of type I collagen by the Serum CrossLaps ELISA (Nordic Bioscience, Denmark). Briefly, the Serum CrossLaps ELISA is based on monoclonal antibodies recognizing the isomerised amino acid sequence EKAHD-β-GGR specific for the C-telopeptide of type I collagen and D-β-G representing the isomerised bond between aspartate and glycine. The sandwich assay which detects cross-linked peptides (CTX-I), has been reported to have intra-assay and inter-assay coefficient of variation of 4.9% and 7.9%, respectively and is useful for the quantitative assessment of bone resorption[34].

### Statistics

Results shown are mean ± SEM. Significant differences between means of study groups were determined using the Student's two-tailed unpaired t-test. Differences were considered statistically significant if p < 0.05.

## Results

### Cathepsin K and CTX-I are expressed in advanced but not early lesions

**Figure 1:**
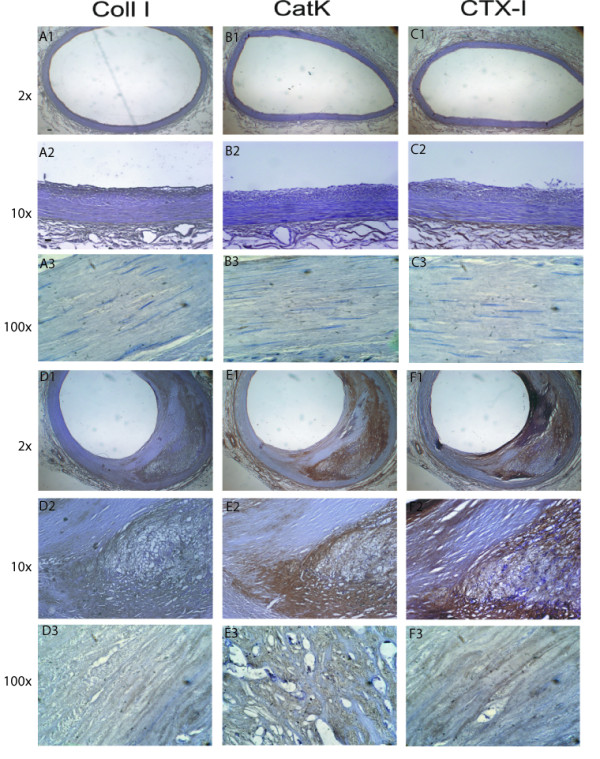
**Immunohistochemical staining of Collagen type I, Cathepsin K, and CTX-I in: early and advanced lesions**. A, B and C: staining of Collagen type I, Cathepsin K and CTX-I, respectively in an early lesion. D, E and F: staining of Collagen type I, Cathepsin K and CTX-I, respectively in an advanced lesion. A1, B1, C1, D1, E1 and F1 are 2× magnified, whereas A2, B2, C2, D2, E2 and F2 are 10 × magnified. Brown colouring suggest positive staining with the particular antibody, whereas blue staining suggest Hematoxyllin staining of the cell nuclei.

Left anterior descending (LAD) coronary arteries with different degrees of atherosclerotic lesions were assessed for the expression and localization of collagen type I, cathepsin K and the CTX-I cleavage product of type I collagen, generated by the action of cathepsin K (Figure 1). Immune-histochemical analysis revealed co-localization of collagen type I and cathepsin K in the advanced atherosclerotic lesions, especially at the plaque shoulders (Figure 1 D1 and 1 E1). In close proximity to the cathepsin staining there was a corresponding accumulation of CTX-I, presumably resulting from the cathepsin K activity on collagen type I. In the relatively unaffected coronary arteries there was little or no positive staining for the cathepsin K and CTX-I (Figure 1 A1, B2 and 1 C1).

### Human monocyte-macrophage foam cells generate CTX-I fragments when cultured on collagen type I-rich matrix

**Figure 2:**
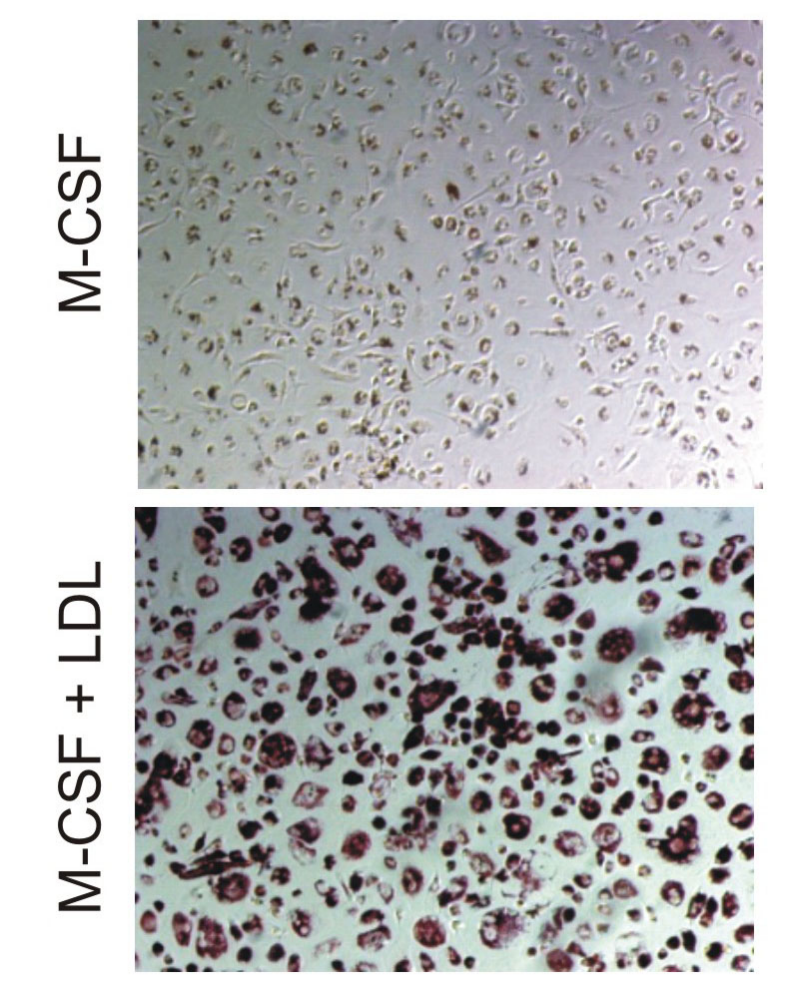
**Culturing of human monocytes and macrophage foam cells**. CD-14 monocytes were isolated and cultured on plastic for 14 days in the presence of either M-CSF alone (Panel A) or M-CSF and 2 mg/ml LDL (Panel B). After the culture, cells were fixed in 4% formaldehyde, and stained for lipid droplets by oil red staining. Monocyte-macrophages cultured in the presence of LDL assumed the appearance of foam cells and were oil-red O positive.

Human monocyte-macrophage cultures were generated using CD-14 positive monocytes cultured on plastic in the presence of M-CSF or M-CSF in combination with LDL. When incubated in the presence of LDL, cells assumed a foam cell like appearance and were oil red positive as illustrated on Figure 2.A and 2.B.

**Figure 3:**
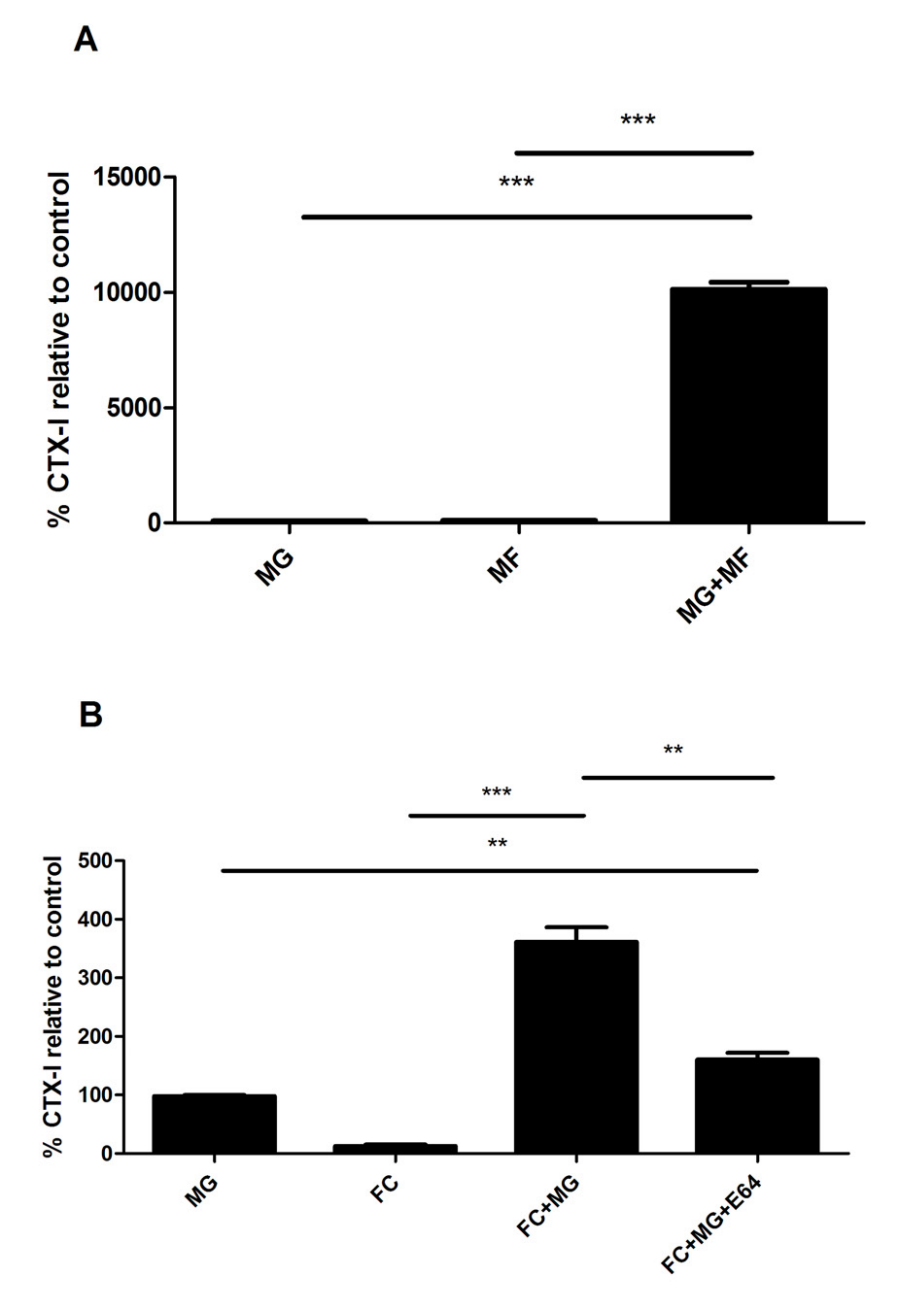
**Cell cultures**. A) Human monocyte-macrophages and B) macrophage-foam cells (FC) were generated and cultured on a matrigel (MG) enriched with 48.8% collagen type I, with or without Cathepsin K inhibitor E64. Collagen type I degradation was measured by CTX-I assay in the conditioned medium after the culture period.

To investigate whether these cells were able to degrade a collagen-rich ECM in part resembling that of the arterial wall, human monocyte-macrophages were generated and cultured on a matrigel (MG) enriched with 48.8% collagen type I. Collagen type I degradation by cathepsin K was measured by assessing the appearance of CTX-I in the conditioned medium after the culture period[23]. As presented in Figure 3A, macrophages (MF) alone did not generate CTX-I fragments above lowest detection limit of the assay. MG alone released to a lesser amount CTX-I fragments. In contrast, MF cultured on MG released 100-fold (p < 0.01) more cathepsin K generated collagen type I fragments (CTX-I), relative to control (matrigel alone). We also assessed the amount of CTX-I released when culturing monocytes in the presence of 2 mg/ml of LDL. Additionally, we also tested the levels of CTX-I released, when cells were cultured in the presence of selective cysteine protease inhibitor - E64. As illustrated in Figure 3B foam cells (FC) did not release any considerable amount of CTX-I when cultured on plastic. Matrigel (MG) released to a lesser amount CTX-I to the culture medium. FC cultured on matrigel released 30-fold (p < 0.01) increased levels of CTX-I relative to MG control. The cysteine protease inhibitor E64, resulted in a similar amount of CTX-I released as in MG control. All cell culture experiments have been validated for cell viability by Alamar blue (data not shown).

### No difference in serum levels of CTX-I in patients and control subjects

Women assigned to the CVD group based on radiographic evidence of aortic calcification had calcification scores between 9 and 24, with an average of 12.6 ± 0.5. This group had higher plasma triglyceride concentrations (p = 0.016) than age-matched control group with no aortic calcification did not differ in systolic blood pressure or total plasma cholesterol, (Table 1).

We assessed serum CTX-I levels in this population to determine if levels might be elevated in subjects with aortic calcification relative to those without, perhaps as evidence of extensive vascular degradation of type I collagen.

**Figure 4:**
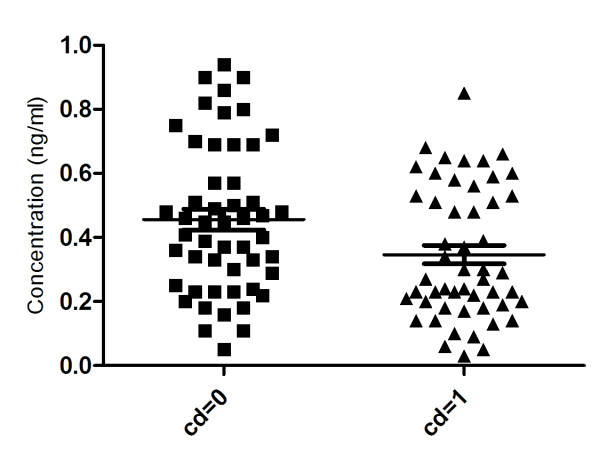
**Levels of CTX-I measured in blood samples for control-group (CD = 0) and disease group (CD = 1)**.

The level of CTX-I was found to be significantly lower in patients with CVD (0.35 ± 0.03 ng/ml) compared to the control group (0.46 ± 0.03 ng/ml), p = 0.014 as presented on Figure 4. Exclusion of individuals receiving anti-resorptive osteoporosis treatments such as bisphosphonates and hormone replacement therapies which lower CTX-I levels by approximately 50% eliminated these differences (0.40 ± 0.04 ng/ml patient group (n = 31), vs. 0.50 ± 0.03 ng/ml (n = 43) control group, p = 0.061). (Figure 4)

## Discussion

Extracellular matrix remodelling is an important process in atherosclerotic plaque turnover and stability. To our knowledge, we are the first to demonstrate that macrophage foam cells generate CTX-I when cultured on collagen type-I rich matrix, providing biological rationale for the co-localisation of CTX-I and cathepsin K in the advanced atherosclerotic lesions.

CTX-I is one of the fragments generated through enzymatic cleavage of collagen type I by cathepsin K[35]. We demonstrated strong positive staining of both cathepsin K and CTX-I in coronary arteries with advanced atherosclerotic plaques in comparison to normal coronary arteries, where cathepsin K staining was very weak. These findings are in accordance with the results previously seen by Lutgens et al[10], demonstrating cathepsin K mRNA expression at low levels in early atherosclerotic lesions which were up-regulated in advanced lesions by up to 28 fold.

As collagen I constitutes 60-70% of the matrix content in arteries[36], we hypothesized that the level of CTX-I would be high [37] in advanced atherosclerotic lesions. Our results from immune-histological stainings of human coronary arteries confirmed this hypothesis and furthermore showed a co-localization of CTX-I and Cathepsin K in the advanced lesion.

To further investigate the biological origin of CTX-I in the advanced atherosclerotic lesions, human macrophage foam cells were generated and cultured on a type I collagen enriched matrix. We used the CD-14 positive selection procedure to have a pure hematopoietic stem cell population, that our group extensively has investigated[19,20]. The aim of these experiments was to investigate whether macrophages and foam cells were able to generate cathepsin K mediated type I degradation products (CTX-I). The role of cathepsin K and foam cells in atherosclerosis has been demonstrated by others[6,7,9,10,21]. We have previously published extensively on the use of osteoclasts and CTX-I[35,38,39], and the use of this hematopoietic cell culture system in bone biology[40]. This work clearly demonstrated that in cell culture, CTX-I was generated by cathepsin K[20,38,41]. This correlated with an increased cathepsin K expression as previously shown [20,33].

The results from the human macrophages and macrophage foam cell cultures clearly demonstrate that human macrophage foam cells produce cathepsin K fragments of collagen type I, the CTX-I. This is to our knowledge this first data describing a simple model system in which human macrophage foam cell mediated degradation of the extra cellular matrix can be monitored. This assay may be used to identify treatments that will modulate the macrophage foam cell degradation of the matrix, by enzymes such as cathepsin K and thus aid in the identification of novel treatments that will attenuate atherosclerotic plaque turnover and subsequently prevent rupture of the unstable plaques.

The measured cathepsin K activity, in form of the generated CTX-I fragment, may arise from both secreted cathepsin K and the cathepsin K in lysosomes. Osteoclasts, a different phenotype of macrophages, have previously been shown to transcytose collagen type I and degraded type I collagen (CTX-I) during bone degradation - and as macrophages are phagocytic cells, a similar pathway involving intracellular lysosome mediated collagen type I degradation may be responsible[42,43]. Further research is needed to differentiate and understand biological differences between these distinct forms of proteolytic activities sequestered in the matrix or lysosomal degradation.

Circulating levels of CTX-I were not elevated in the population of largely post-menopausal women suffering from CVD. This observation is likely explained by the dominating contribution of CTX-I derived from bone-resorption [44] which would be expected to be relatively high in post-menopausal women not on anti-resorptive therapies. This is supported by the observation that exclusion of the sub-group of women receiving hormone and osteoporosis treatment abolished apparent differences in the circulating CTX-I levels between the groups. Anti-osteoporosis treatment are known to lower CTX-I levels by approximately 50%, explaining this observation[23]. Local proteolytic events in the atherosclerotic plaques seem to be associated with plaque instability and plaque rupture, and not directly with the amount of arterial calcification. However, as the number and extend of calcifications are directly proportional to the ODDs ratio of experiencing a CVD event, there might be an indirect relation[45]. Arterial calcification is an end-stage of actively remodeling plaques, which either results in rupture or become stable plaques.

The group of patients with diagnosed CVD had an average AC-score of 12.6. We demonstrated significantly higher levels of triglyceride in CVD-group compared to the control group. As shown by previous studies there is a correlation between the level of triglyceride and the risk of CVD [46-49]. Thus, even though that the patient population may be appropriate for detecting an increased level of CTX-I related to the arterial pathogenesis, this was not possible, which most likely is due to the contribution of bone related CTX-I to the systemic pool.

One limitation of the current study is the variation in induction of the amount of CTX-I generated in cultures with macrophages and macrophage-foam cells. These variations are most likely due to experimental variation of batch numbers of proteins and matrigel, as well as variation in cell number. Further research is needed for obtaining a more robust system, which will allow for careful investigation of pathophysiological processes mediated by macrophages.

## Conclusions

In conclusion, our data suggest, that CTX-I might be a specific marker of pathophysiological processes involved in the extracellular remodeling of plaques. Cathepsin K and CTX-I were absent from early human atherosclerotic lesions while being present in advanced lesions, suggesting this class of biomarkers as useful tools for diagnosing and staging of the atherosclerotic lesions. The herein described assay and techniques may aid others researchers in the identification of important pathways leading to increased levels of proteolytic activity on macrophages, and thus progress the development of the identification of novel treatment of atherosclerosis.

## Competing interests

Morten Karsdal and Claus Christiansen hold stock in Nordic Bioscience. Other authors have no competing interests.

## Authors' contributions

NB conceived the study together with MAK, and participated in its design and coordination. Furthermore, NB carried out immune-histochemistry analysis, data analysis on human samples and cell cultures. HSA, performed some of the macrophage cell-culture experiments and participated in the data analysis. TCR, drafted the manuscript and provided samples for immune-histochemistry. LL, performed ELISA assay measuring CTX-I and cultured macrophage foam cells. IB performed statistical analysis and participated in the data analysis. CC drafted the manuscript and provided human samples for biomarker measurements. MAK participated in overall study design and planning, data analysis, discussions and conclusions. All authors read and approved the manuscript.

## Pre-publication history

The pre-publication history for this paper can be accessed here:

http://www.biomedcentral.com/1471-2261/10/19/prepub
